# Left ventricular ejection fraction and left atrium diameter related to new-onset atrial fibrillation following acute myocardial infarction: a systematic review and meta-analysis

**DOI:** 10.18632/oncotarget.20821

**Published:** 2017-09-11

**Authors:** Rui-Xiang Zeng, Mao-Sheng Chen, Bao-Tao Lian, Peng-Da Liao, Min-Zhou Zhang

**Affiliations:** ^1^ Division of Chest Pain Center, Guangdong Provincial Hospital of Chinese Medicine, The 2nd Clinical College of Guangzhou University of Chinese Medicine, Guangzhou, 510120, P.R. China

**Keywords:** atrial fibrillation, left ventricular ejection fraction, left atrium diameter, acute myocardial infarction

## Abstract

**Background:**

New-onset atrial fibrillation (NOAF) occurs frequently in patients with acute myocardial infarction (AMI), and is associated with increased subsequent cardiovascular mortality. However, only a few studies directly evaluated the relationship of left ventricular ejection fraction (LVEF) or left atrium diameter (LAD) and NOAF following AMI.

**Materials and Methods:**

MEDLINE^®^, EMBASE^®^ and the Cochrane Library were carried out to find studies until January 2017. Pooled mean difference (MD) and 95% confidence interval (CI) were calculated to evaluate the value of LVEF and LAD in the prediction of NOAF after AMI. We performed sensitivity analyses to explore the potential sources of heterogeneity. Statistical analyses were carried out using the Revman 5.3.

**Result:**

We included 10 qualifying studies comprising a total of 708 patients with NOAF and 6785 controls. Overall, decreased LVEF and increased LAD levels had a significant positive association with NOAF in patients with AMI. The MD in the LVEF levels between the patients with and those without NOAF was −4.91 units (95% Cl: −5.70 to −4.12), test for overall effect z-score = 12.18 (*p* < 0.00001, I^2^ = 35%). Moreover, in a subgroup analysis, the MD for LAD and NOAF was 2.55 units (95% Cl: 1.91 to 3.19), test for overall effect z-score = 7.80 (*p* < 0.00001, I^2^ = 57%).

**Conclusions:**

Our meta-analysis demonstrated that both decreased LVEF and increased LAD levels were associated with greater risk of NOAF following AMI.

## INTRODUCTION

Atrial fibrillation (AF) occurs commonly in hospitalized patients with acute myocardial infarction (AMI), with a reported incidence between 2% and 20% [1], and is closely associated with prolonged hospitalization, increased subsequent cardiovascular mortality in AMI patients [2–6]. The development of AF in the AMI setting is multiple factors, including older age, systemic inflammation, heart failure, acute ischemia, elevated left ventricular (LV) end-diastolic pressure, left atrial (LA) enlargement or infarction [7]. As well known, left ventricular ejection fraction (LVEF) serves as a significant prognostic marker of cardiac function, and left atrium diameter (LAD) responses to whether left atrial enlargement or not, both of those abnormalities are considered as a risk predictor for cardiovascular disease. However, to our knowledge, only a few studies directly evaluated the associations between LVEF or LAD and new-onset AF (NOAF) in patients with AMI. So we conducted this comprehensive meta-analysis to explore the impact of LVEF on NOAF following AMI by collecting data for previously published studies. Furthermore, the relationship of LAD and NOAF was assessed by a subgroup analysis.

## MATERIALS AND METHODS

### Literature search

Our study strictly complies with the guidelines of the meta-analysis of observational studies in epidemiology group (MOOSE) [8]. A comprehensive systematic search of MEDLINE^®^, EMBASE^®^ and the Cochrane Library was carried out to find relevant studies until January 2017. Searches combined free-text and MeSH terms relating to “left ventricular ejection fraction” or “LVEF,” “atrial fibrillation” and “myocardial infarction” or “myocardial infarct”. Reference lists from the identified articles were manually examined for relevant new articles. Abstracts, unpublished reports, and non-English language articles were not included.

### Inclusion and exclusion criteria

The inclusion criteria were as follows: 1) the study had the baseline LVEF levels data based on with and without NOAF after AMI; and 2) the study used NOAF rates as an outcome. The exclusion criteria were: 1) history of AF and did not focus on AMI; 2) lacked of preprocedural LVEF levels; 3) Abstracts without the full text.

### Identification of studies

We restricted our search to studies published in English. Abstracts and titles of related articles were initially scanned by a reviewer. Potentially relevant articles were then considered by at least 2 independent reviewers. Disagreements were resolved by discussion or upon consensus from a 3rd or 4th reviewer. Two reviewers agreed on the inclusionary or exclusionary status of 90% of the reviewed studies. Full texts of the selected articles were then screened by both authors for inclusion in the review. All disagreements were resolved by consensus.

### Quality assessment and data extraction

Quality assessments were evaluated with the Newcastle-Ottawa Scale (NOS) list for nonrandomized studies. Each study was assessed in three aspects using this “star system”: the selection of the study groups; the comparability of the groups; and the ascertainment of the outcome of interest (Supplementary Tables 1 and 2).

Two blinded reviewers independently used a standardized data-extraction form to determine appropriately to extract data. We extracted data included the lead author's last name, the publication year, and the origin of the studied population; the study design; the characteristics of the studied population (sample size, age, sex, time of AF detection, and withdrawals and dropouts of patients); endpoint evaluations (definitions of NOAF and methods of AF detection); rates of NOAF; and means and SDs of LVEF in each group. Disagreements were resolved by consensus from another reviewer.

### Statistical analysis

The association strength between LVEF or LAD and NOAF was measured by the mean difference (MD) and 95% confidence interval [CI). The significance of pooled MD was tested by *z*-test (*P* < 0.05 was considered significant). Heterogeneity was evaluated with Cochran's Q statistic and quality by I^2^ statistic. We premeditated that mild heterogeneity might be less than 30 percent of the variability in point estimates and the values of I^2^ exceeding 50% might express as significant heterogeneity [9], so we used the random-effects model for our study and between study variance, otherwise, with a fixed-effects model. To explore sources of heterogeneity, we performed several sensitivity analyses. Publication bias was also evaluated by inspecting funnel plots. All analyses were conducted with the use of Review Manager, version 5.3 (Revman, The Cochrane Collaboration; Oxford, UK).

## RESULTS

### Search results

**Figure 1:**
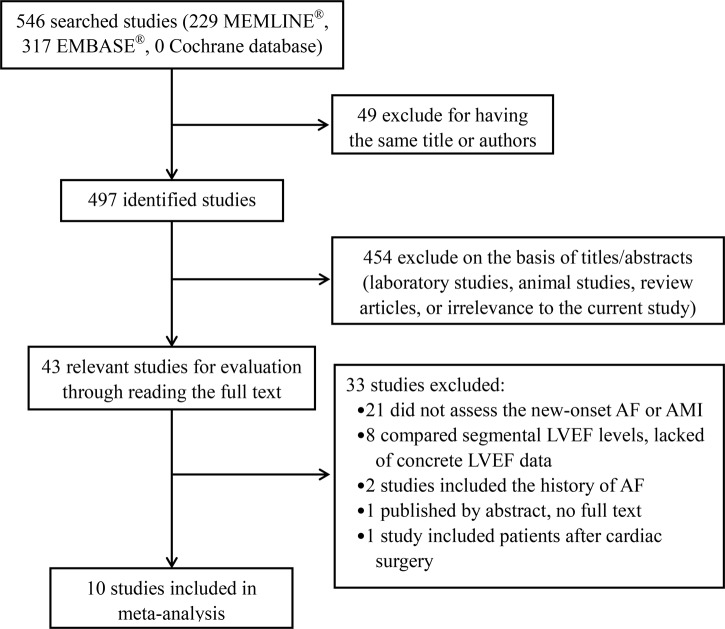
Flow diagram of the trial-selection process AF = atrial fibrillation; AMI = acute myocardial infarction; LVEF = left ventricular ejection fraction.

The search yielded 546 research reports, of which 49 were excluded for having the same title or authors; 454 were excluded because they were laboratory studies, animal studies, review articles, or irrelevance to the current analysis. Of the remaining 33 studies, 21 studies did not assess the NOAF or AMI. 8 studies researched segmental LVEF levels and lacked of concrete LVEF data. 2 studies included the history of AF. One study just published by abstract. One study included patients after cardiac surgery. The foregoing studies were all excluded, and 10 observational studies [10–19] were finally included in our meta-analysis (Figure 1). As a result, 7493 patients were involved in our analysis: 708 patients in AF group and 6785 patients in without AF group.

### Baseline characteristics and quality assessment

The NOS for assessing the quality of the 10 studies is shown in Table 1 and the scores ranged from 6–7. Table 2 presents the characteristics of each study. The average age of patients in the included studies ranged from 58 to 74 years and the rate of NOAF ranged from 7.44% to 20.7%.

**Table 1:** Evaluation of the quality of the 10 included studies by using the Newcastle-Ottawa Scale^#^

Factor	Study type	Selection				Comparability	Exposure or outcome			No. of star
		1	2	3	4		1	2	3	
Cicek 2003	Case-Control Study	*	*	*	*	*	*	*		7
Aronson 2007	Cohort Study	*	*	*	*	*	*	*		7
Gedikli 2008	Case-Control Study	*	*	*	*	*	*	*		7
Bahouth 2009	Cohort Study	*	*	*	*	*	*	*		7
Hwang 2011	Case-Control Study	*	*	*	*	*	*	*		7
Aronson 2011	Cohort Study	*	*	*	*	*	*	*		7
Yoshizaki 2012	Case-Control Study	*	*	*	*	*	*	*		7
Dorje 2013	Case-Control Study	*	*	*	*	*	*	*		7
Guenancia 2014	Case-Control Study	*	*	*		*	*	*		6
Zhang 2014	Case-Control Study	*	*	*	*	*		*		6

^#^The Newcastle-Ottawa Scale criteria are listed in supplementary files.

**Table 2:** Characteristics of the 10 studies included in the meta-analysis

Factor	Year	Study population	Patients, n	Male, n	Mean Age, yrs	New-onset AF rate, %	Time of AF detection	Timing of LVEF determination	Methods of AF detection	Method of revascularization
Cicek D, et al.	2003	Turkey	100	77	59	19%	During hospitalization	N/A	ECG was monitored continuously	Thrombolytic (69%)
Aronson D, et al.	2007	Israel	1209	936	62	11.3%	During the index hospitalization	Pre-interventional	Telemetry strips and ECGs	N/A
Gedikli O, et al.	2008	Turkey	92	67	58	20.7%	During the first 7d after AMI	N/A	ECG was monitored continuously	N/A
Bahouth F, et al.	2009	Israel	1920	1505	64	8.4%	At admission or later during the hospital stay	A median of 2 days from admission	Telemetry strips and ECGs	N/A
Hwang HJ, et al.	2011	South Korea	401	294	61	8.2%	Within 24 h after AMI	Pre-interventional	Telemetry strips and ECGs	PTCA, CABG or medical treatment
Aronson D, et al.	2011	Israel	1169	817	64	9.4%	During a follow-up period of 6 months.	A median of 2 days from admission	Telemetry strips and ECGs	N/A
Yoshizaki T, et al.	2012	Japan	176	152	74	13.6%	During hospitalization	On day 5–7 of admission	ECG was monitored continuously	N/A
Dorje T, et al.	2013	China	268	224	64	13.4%	During the AMI hospitalization	Pre-interventional	Telemetry strips and ECGs	N/A
Guenancia C, et al.	2014	France	1123	779	79	8.1%	During the AMI hospitalization	On admission	Telemetry strips and ECGs	PCI (69%) Other (31%)
Zhang X, et al.	2014	China	1035	693	65	7.44%	During the AMI hospitalization	Pre-interventional	ECG was monitored continuously	PCI (23.38%) Thrombolysis (1.30%)

AF = atrial fibrillation; LVEF = left ventricular ejection fraction; ECG = electrocardiograph; N/A = not applicable; AMI = acute myocardial infarction; PTCA = percutaneous transluminal coronary angioplasty; CABG = coronary artery bypass grafting; PCI = percutaneous coronary intervention.

### Quantitative data synthesis and heterogeneity analysis

**Figure 2:**
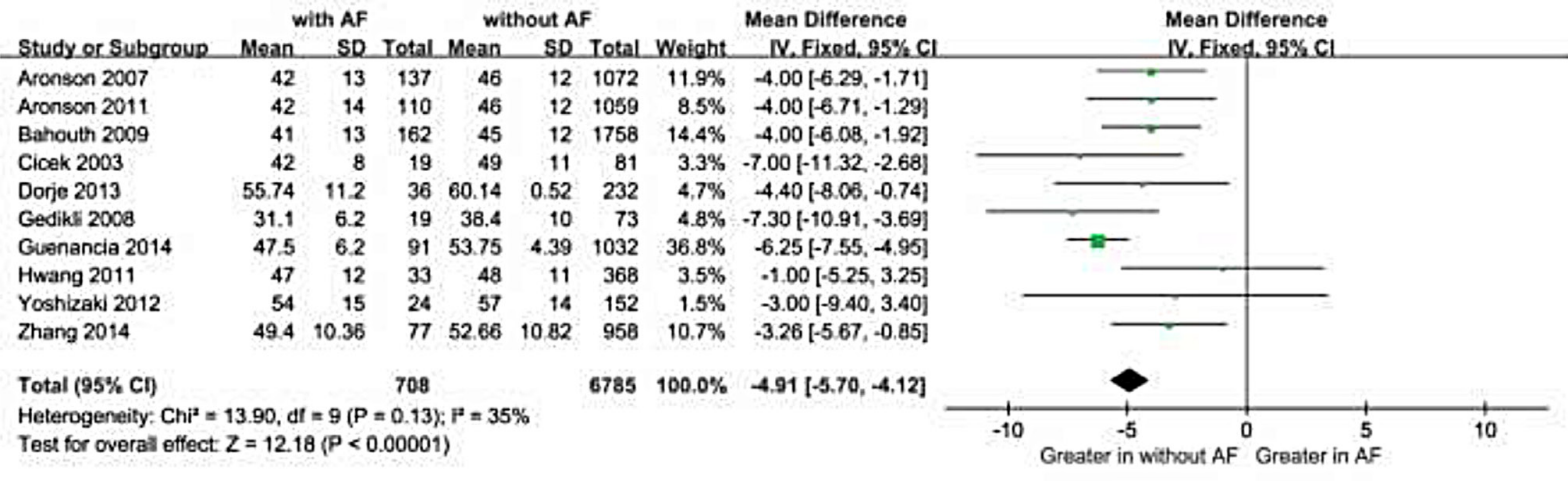
Comparison of LVEF levels between AF and without AF groups in the 10 included studies CI = confidence interval; AF = atrial fibrillation; LVEF =left ventricular ejection fraction.

**Figure 3:**
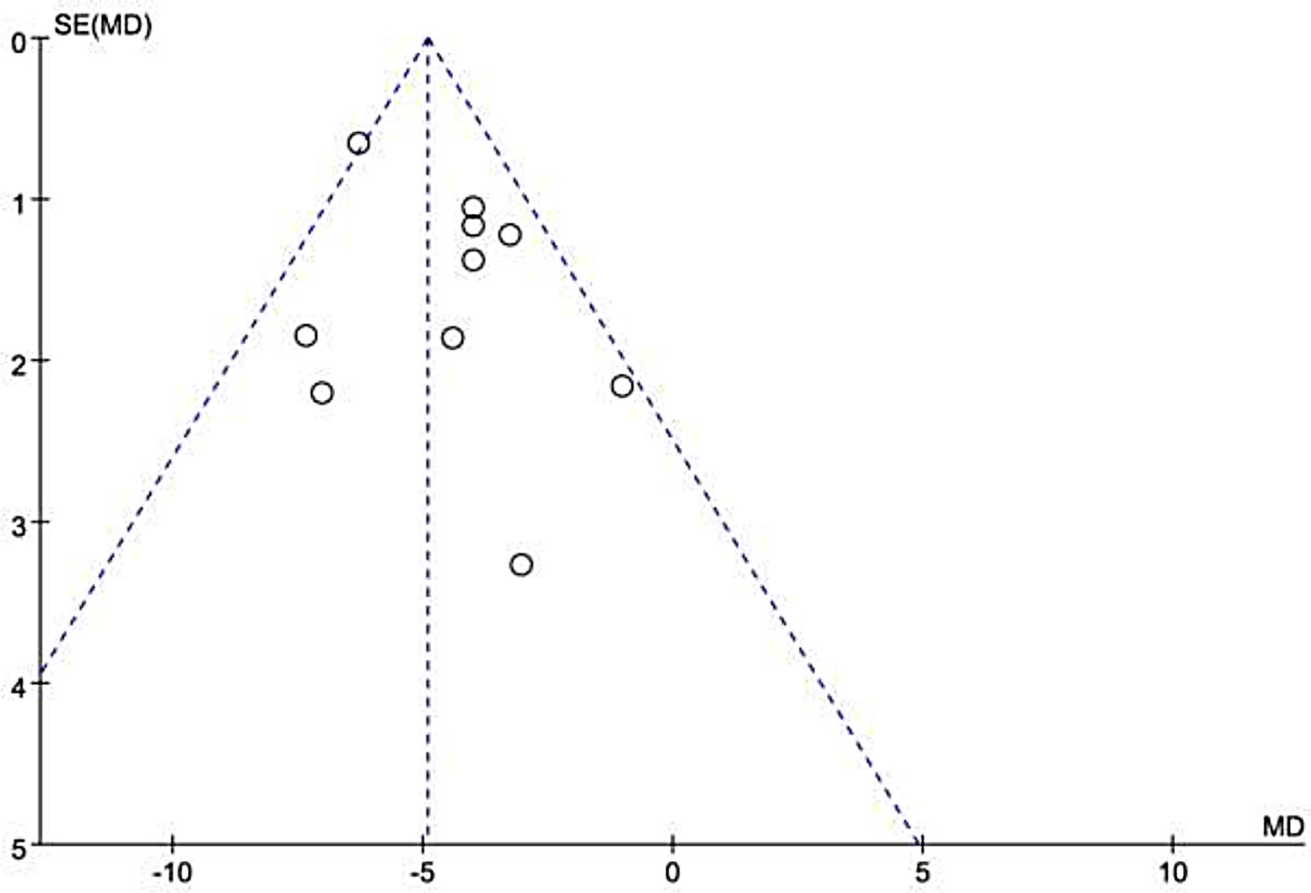
Funnel plot of the 10 included studies SE = standard error; MD = mean difference.

Overall, decreased baseline LVEF levels had a significant positive association with NOAF in patients with AMI. The MD in the LVEF levels between the patients with and those without NOAF was −4.91 units (95% Cl: −5.70 to −4.12), test for overall effect z-score = 12.18 (*p* < 0.00001, I^2^ = 35%) (Figure 2). However, an asymmetric funnel plot showed the possible existence of publication bias (Figure 3). Because of small sample size, we could not explain the exact cause of heterogeneity in our meta-analysis.

**Figure 4:**
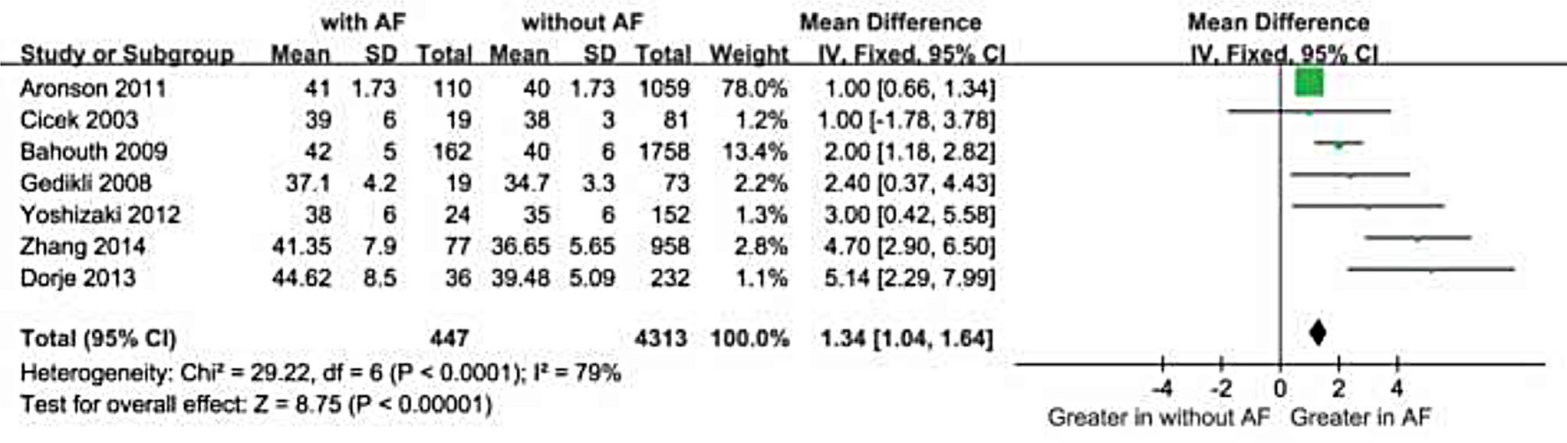
Comparison of LAD levels between AF and without groups in the 7 included studies CI = confidence interval; AF = atrial fibrillation; LAD = left atrium diameter.

**Figure 5:**
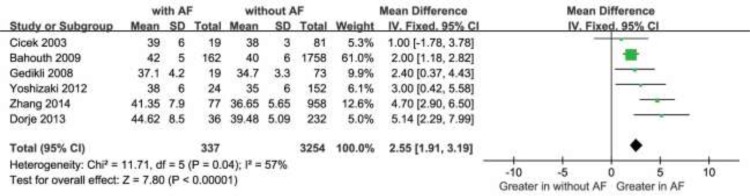
Comparison of LAD levels between AF and without groups in the remaining 6 included studies CI = confidence interval; AF = atrial fibrillation; LAD = left atrium diameter.

Moreover, The MD in a subgroup analysis for LAD levels between the patients with, and those without NOAF was 1.34 units (95% Cl: 1.04 to 1.64), test for overall effect z-score = 8.75 (*p* < 0.00001, I^2^ = 79%) (Figure 4). The heterogeneity test showed that there were significant differences between individual studies (*p* < 0.00001; I^2^ = 79%). We subsequently performed sensitivity analyses in order to identify the origin of this heterogeneity [20]. As shown in Figure 5, after excluding the studies by Aronson D et al. [19] the heterogeneity test showed less effects on the results (*p* < 0.00001, I^2^ = 57%), whereas the MD in the LAD levels between the patients with and without NOAF was 2.55units (95% Cl: 1.91 to 3.19), test for overall effect z-score = 7.80 (*p* < 0.00001). As known, the study by Aronson D et al. [19] had a longer follow-up period of 6 months, which was different from the remaining 6 studies, and this might be a possible source of heterogeneity.

## DISCUSSION

Interestingly, our meta-analysis demonstrated that lower LVEF levels were associated with NOAF occurrence after AMI. Furthermore, in a subgroup analysis, we also found that increased LAD levels related to greater risk of NOAF following AMI, although there was significant heterogeneity. Nonetheless, sensitivity analyses indicated that differences in follow-up period might account for the heterogeneity. Thus, present study might provide insights into mechanisms and lead to greater understanding of the risk factors for NOAF after AMI.

As well known, current evidence regarding the associations between NOAF and in-hospital or long-term outcomes in AMI patients is convincing. These outcomes include the length of hospital stay, heart failure, stroke, recurrent myocardial ischemia, major bleeding and increased mortality [5, 21–25]. Therefore, prediction of NOAF following hospitalization for AMI may reduce clinical adverse events [26]. In the setting of AMI, previous studies have demonstrated a number of risk factors for NOAF in AMI patients, such as old age, female gender, obesity, history of hypertension, history of stroke, higher Killip class or heart failure, hypotension, higher heart rate, higher CHADS2 score, increased peak creatinine kinase, C-reactive protein and N-terminal pro-brain natriuretic peptide levels [5, 6, 13, 16, 21, 23–25, 27, 28]. To our knowledge, this is the first meta-analysis directly evaluating the impact of LVEF and LAD on NOAF in patients with AMI.

Even though the exact mechanism for LVEF or LAD in AMI patients with NOAF was still unclear, several previous relevantly studies have contributed evidence to investigate it and provided potential responsible mechanisms. Aronson and colleagues reported that both LVEF and LAD were independently associated with NOAF, suggesting that increased LV filling pressures may contribute to the development of AF after AMI [19]. Numbers of studies have reported multitudinous pathologic mechanisms of AF following AMI, which could include abrupt changes such as increased LV filling pressure, deterioration of LV systolic functions, or direct ischemic insult of the atria [11, 22, 23, 29–32]. AMI often results in change LV filling dynamics, which may lead to advanced diastolic dysfunction. Subsequently, diastolic dysfunction may produce increased LA pressure and initiate LA remodeling, promoting the progression to AF [5, 7, 19]. In addition, experimental and clinical researches have demonstrated that increasing atrial pressure and/or causing acute atrial dilatation may act an important part in the development of AF in the AMI [5–7, 18, 29, 33]. Hence, it was not difficult to understand that left atrial enlargement that assessed by LAD was major predisposing factors for the development of AF [34–36]. Overall, all of above might indicate the potential mechanisms for the result of the present meta-analysis.

From the conclusion of this study, we could deduce that decreased LVEF and increased LAD levels might be associated with worse clinical prognosis in patient with NOAF following AMI. Early identification of patients with AMI who are at risk of AF attack is of particular importance. Hence, NOAF should be close monitoring for avoiding hemodynamic depression. Management of AF in patients with AMI should follow guideline recommendations [37–39]. It has been well established that oral anticoagulation is a proven therapy for stroke prevention in AF patients with high thromboembolic risk [21]. However, there is insufficient evidence to support prophylactic anticoagulant therapy for AMI patients with high risk of AF. Further studies specific to AF prevention in the patient with AMI are needed.

Our meta-analysis may provide worthy and reliable information regarding the relationships between LVEF, LAD, and NOAF in patients with AMI. However, there are still some potential limitations to this meta-analysis. First, the definitions of NOAF and methods of AF detection were not accordant, and some information on the timing of LVEF determination and the method of revascularization was not applicable, which might be subject to the source of potential bias. Second, most of the included studies did not directly research the impact of LVEF and/or LAD on NOAF after AMI, some potential confounders might have not entirely eliminated. Third, our analysis was based on observational studies, and the numbers of studies and patients were rather limited. Finally, the conclusions of the absence of publication bias were not always reliable. Therefore, the results of our analysis should be interpreted cautiously, and future investigations are needed to clarify the mechanisms of NOAF further.

## CONCLUSIONS

In conclusion, our meta-analysis demonstrated that both decreased LVEF and increased LAD levels might be associated with greater risk of NOAF following AMI, which contributing compelling evidence that LVEF and LAD may be a useful marker in predicting NOAF in AMI patients.

## SUPPLEMENTARY MATERIALS TABLES


