# Supplementary material for: Left ventricular ejection fraction and left atrium diameter related to new-onset atrial fibrillation following acute myocardial infarction: a systematic review and meta-analysis
Source: Oncotarget. 2017 Sep 11;8(46):81137–44. doi: 10.18632/oncotarget.20821 (PMC5655268; doi:10.18632/oncotarget.20821)
Supplement: Supplementary file 1 [file oncotarget-08-81137-s001.pdf]

Left ventricular ejection fraction and left atrium diameter related to new-onset atrial fibrillation following acute myocardial infarction: a systematic review and meta-analysis

SUPPLEMENTARY MATERIALS

Supplementary Table 1: Newcastle-Ottawa quality assessment scale–cohort studies

Variable	Possible Response
**Selection**	
1. Representativeness of the exposed cohort	a) truly representative of the average ________ (describe) in the community*<br>b) somewhat representative of the average _______ in the community*<br>c) selected group of users eg nurses, volunteers<br>d) no description of the derivation of the cohort
2. Selection of the non-exposed cohort	a) drawn from the same community as the exposed cohort*<br>b) drawn from a different source<br>c) no description of the derivation of the non-exposed cohort
3. Ascertainment of exposure	a) secure record (eg surgical records)*<br>b) structured interview*<br>c) written self report<br>d) no description
4. Demonstration that outcome of interest was not present at start of study	a) yes*<br>b) no
**Comparability**	
Comparability of cohorts on the basis of the design or analysis	a) study controls for _____ (select the most important factor)*<br>b) study controls for any additional factor (This criteria could be modified to indicate specific control for a second important factor.)*
**Outcome**	
1. Assessment of outcome	a) independent blind assessment*<br>b) record linkage*<br>c) self report<br>d) no description
2. Was follow-up long enough for outcomes to occur	a) yes (select an adequate follow up period for outcome of interest)*<br>b) no
3. Adequacy of follow up of cohorts	a) complete follow up - all subjects accounted for*<br>b) subjects lost to follow up unlikely to introduce bias - small number lost->__% (select an adequate%) follow up, or description provided of those lost)*<br>c) follow up rate <__% (select an adequate %) and no description of those lost<br>d) no statement

A study can be awarded a maximum of one star for each numbered item within the Selection and Outcome categories. A maximum of two stars can be given for Comparability.

Supplementary Table 2: Newcastle-Ottawa quality assessment scale–case control studies

Variable	Possible Response
**Selection**	
1. Is the case definition adequate?	a) yes, with independent validation*<br>b) yes, eg record linkage or based on self reports<br>c) no description
2. Representativeness of the cases	a) consecutive or obviously representative series of cases*<br>b) potential for selection biases or not stated
3. Selection of Controls	a) community controls*<br>b) hospital controls<br>c) no description
4. Definition of Controls	a) no history of disease (endpoint)*<br>b) no description of source
**Comparability**	
Comparability of cohorts on the basis of the design or analysis	a) study controls for _____ (select the most important factor)*<br>b) study controls for any additional factor (This criteria could be modified to indicate specific control for a second important factor.)*
**Exposure**	
1. Ascertainment of exposure	a) secure record (eg surgical records)*<br>b) structured interview where blind to case/control status*<br>c) interview not blinded to case/control status<br>d) written self report or medical record only<br>e) no description
2. Same method of ascertainment for cases and controls	a) yes*<br>b) no
3. Non-Response rate	a) same rate for both groups*<br>b) non respondents described<br>c) rate different and no designation

A study can be awarded a maximum of one star for each numbered item within the Selection and Exposure categories. A maximum of two stars can be given for Comparability.
